# Does Faux Pas Detection in Adult Autism Reflect Differences in Social Cognition or Decision-Making Abilities?

**DOI:** 10.1007/s10803-015-2551-1

**Published:** 2015-08-15

**Authors:** Flora I. Thiébaut, Sarah J. White, Annabel Walsh, Solja K. Klargaard, Hsuan-Chen Wu, Geraint Rees, Paul W. Burgess

**Affiliations:** Institute of Cognitive Neuroscience, University College London, 17 Queen Square, London, WC1N 3AR UK; Wellcome Trust Centre for Neuroimaging, University College London, London, UK

**Keywords:** Autism spectrum disorder, Faux pas, Social cognition, Decision making, Open-ended, Compensatory strategy, Executive function

## Abstract

43 typically-developed adults and 35 adults with ASD performed a cartoon faux pas test. Adults with ASD apparently over-detected faux pas despite good comprehension abilities, and were generally slower at responding. Signal detection analysis demonstrated that the ASD participants had significantly greater difficulty detecting whether a cartoon depicted a faux pas and showed a liberal response bias. Test item analysis demonstrated that the ASD group were not in agreement with a reference control group (n = 69) about which non-faux pas items were most difficult. These results suggest that the participants with ASD had a primary problem with faux pas detection, but that there is another factor at work, possibly compensatory, that relates to their choice of a liberal response criterion.

## Introduction

Autism is a neurodevelopmental disorder involving impairments in social interaction and communication, and restricted, repetitive patterns of behaviour, interests or activities (DSM-5, American Psychiatric Association 2013; ICD-10, World Health Organization 1992). One common explanation for the difficulties in social interaction is a deficit in the ability to mentalize, or Theory of Mind (ToM), the aptitude for inferring other people’s states of mind, such as intentions, beliefs, desires and wishes (Frith and Frith 2006). While older and more high-functioning individuals on the autism spectrum tend to pass traditional ToM tasks used with lower-functioning children, they display persistent daily difficulties understanding other people’s states of mind (Frith et al. 1994).

In order to reveal these individuals’ persistent ToM deficits, Baron-Cohen and colleagues developed an advanced ToM task based on the ability to recognise faux pas (Baron-Cohen et al. 1999). A faux pas was defined as a situation where “a speaker says something without considering if it is something that the listener might not want to hear or know, and which typically has negative consequences that the speaker never intended” (Baron-Cohen et al. 1999, p. 408). The recognition of a faux pas is considered an advanced test of ToM ability as it requires subtle social reasoning: one must be able to appreciate (a) that two protagonists might have different knowledge states and also (b) the emotional impact a statement can have on the listener (Baron-Cohen et al. 1999; Lee et al. 2010). In this way, recognition of a faux pas committed by others is closely related to recognition of embarrassment; the Oxford Dictionary defines a faux pas as “an embarrassing or tactless act or remark in a social situation” (Faux pas [Def. 1] 2015). By definition therefore, all faux pas statements lead to an awkward situation where one or more character is embarrassed. Indeed, all those involved in a particular situation who realise a faux pas has occurred tend to feel embarrassed: the person who committed the faux pas, the person who was affected by it, and any witnesses.

High-functioning children and adults with autism spectrum disorder (ASD) who perform well on first- and second-order false-belief tasks consistently display difficulties in recognising faux pas situations (Baron-Cohen et al. 1999; Zalla et al. 2009). In Baron-Cohen’s study, the group of children with ASD showed a tendency to under-detect faux pas in comparison to a control group (Baron-Cohen et al. 1999). Interestingly, adults with medial prefrontal cortex (mPFC) lesions also tend to under-detect faux pas (Stone et al. 1998); this region is both widely associated with ToM (Frith and Frith 2006) and known to be abnormally recruited by people with ASD (Gilbert et al. 2008). Surprisingly however, a recent study testing adults with ASD on an adapted adult version of the same faux pas task (Stone et al. 1998) found the opposite pattern (Zalla et al. 2009): adults with ASD tended to over-detect faux pas, thinking that they had occurred when in fact they had not.

In this study, we examine four possible explanations for this apparent “over-detection” of faux pas in adults. The first possibility (Hypothesis 1) is that a feature of autism might be excessive attribution of mental states, i.e. to over-mentalizing. This seems prima facie unlikely since ToM impairments are widely attributed to a *lack* of attribution and understanding of others’ mental states, i.e. to under-mentalizing (Frith 2004). However, it is a possibility that should nevertheless be examined in a study of this kind. If this account were true, then the ASD participants would be likely, when presented with faux pas test-like formats, to always detect faux pas when they are present, but also over-detect, perhaps, when they are not. Moreover, their performances should be well predicted by the difficulty of the items in controls—i.e. if typically developing (TD) adults find a faux pas is easy to detect then they will detect it easily too, and similarly, if a faux pas is hard to detect, then they should also find it hard, even if they then may adopt a liberal criterion for deciding that one is present (i.e. require less evidence before deciding that a faux pas has been committed). However, their performance overall would be expected to be good, and in line with TD adults who similarly adopt a liberal criterion.

A second, putative account (Hypothesis 2) is that, as a consequence of poor mentalizing skills, adults with ASD compensate by becoming over-sensitive to embarrassment; adults with ASD are certainly capable of experiencing vicarious social pain (Paulus et al. 2013), although their affective responses to vicarious embarrassment may be modulated and reduced by their difficulties in understanding and integrating another person’s mental state. Even children with ASD seem to have a rather good conceptual understanding of embarrassment (Capps et al. 1992; Hillier and Allinson 2002a, b). It is possible therefore that the combination of poor mentalizing ability plus intact awareness of embarrassment might lead an individual with ASD to be over-sensitive to potentially embarrassing situations (Hypothesis 2a). A strongly related version of this (Hypothesis 2b) is that, having been told, or having learnt through experience, that they are poor in such situations, people with ASD deliberately adopt a strategy of suspecting embarrassment potential when in doubt, but this is not due to mentalizing difficulties. These accounts both predict adoption of a liberal criterion for saying that a faux pas has been committed, but (2a) also predicts poor ability to detect faux pas when they are present. An account of these “increased sensitivity” types may also predict relatively fast reaction times (RTs) when faux pas are presented, because the ASD participants are, in effect, primed to see them.

A third possible explanation (Hypothesis 3) for “false positives” in adult ASD participants’ faux pas responses encompasses a variety of hypotheses that can be loosely grouped together as all involving social cognitive processes. One example is that knowing when a faux pas has *not* been committed is a harder form of social judgement than detecting a faux pas when it has been committed. This may occur for instance if detection of faux pas proceeds through a trial-and-error process of attempting to fit a set of experience-based social schemas of “embarrassing situations” to the stimuli. Where no faux pas is depicted in the stimulus materials, the fitting or search process will on average be more extensive (i.e. because it will have to run until exhaustion) than where a faux pas is shown. If the problem that people with ASD have with performance of faux pas tests is because they have a decrement in a “social cognition” mental resource, and individual variation in this same resource is also the cause of performance differences between TD individuals, then the test items that TD adults find hard (or easy) should also be found relatively hard (or easy) by people with ASD. In other words, the mean performance or intercept may change, but the relative difficulties (as measured by accuracy) of different test items should be similar across the two populations. This hypothesis predicts that RTs should be slower when stimuli are being shown that contain no faux pas, since the exhaustive searching and problem-solving that will be required to decide that there was no faux pas will be reflected in response times.

A fourth possible explanation (Hypothesis 4) concerns non-social decision-making processes and makes a very different prediction. Specific cognitive processes are recruited when dealing with “open-ended” (or in the jargon of the field “ill-structured”) situations that are not also involved when one is dealing with well-structured problems (Burgess et al. 2007). Open-ended problems have a typical set of characteristics, for example, (a) there may be many ways to achieve a given aim; (b) participants have to decide for themselves what constitutes success; (c) success or failure is not clearly signalled at the time of problem-solving. It is easy to see how a test item that asks whether a faux pas has been committed when it does not actually depict one (a “non-faux pas item”) may differ along this dimension of “open-endedness” compared with an item that requires detection of a faux pas when one is depicted. For instance, when one has detected a faux pas, one can be fairly sure of the correctness of one’s response. However, when one responds that no faux pas is depicted, there will always be the possibility that one exists but it was not detected (and therefore that one should have carried on looking). The participant has to set the criteria for their decision point themselves, which is one of the characteristics of “open-endedness”. People with a diagnosis of ASD tend to be poorer at open-ended neuropsychological tasks compared with well-structured ones (White et al. 2009). So it is plausible that non-faux pas items may be harder for people with ASD than faux pas ones quite independently of their social content. This possibility predicts a specific pattern of results on non-faux pas items by individuals with ASD: if the problem with these items is independent of the social content of the items, then item-by-item variability in performance will not match that of TD adults who will have no difficulties dealing with open-ended situations. In this circumstance, most of the variance in TD data will reflect the difficulty of the social processing of the items, but the variance in the ASD non-faux pas items will reflect individual differences in ability to deal with open-ended situations. Thus item-by-item accuracies should be similar between different samples of the TD population, but these values should not be well predictive of item-by-item accuracies in an ASD population. Furthermore, on the faux pas items (i.e. where a faux pas is depicted) ASD participants’ item-by-item performance should be relatively closer to the TD population, since they should find items that are less “open-ended” but require a considerable depth of social processing hard in a similar way to TD populations. This account should also predict that RTs to the non-faux pas items should be slower in ASD participants than they are for faux-pas items, since it is implausible that problems dealing with the open-endedness of the non-faux pas items would not have a consequence for processing and decision speed.

These four possibilities were investigated in this study. We used a newly created version of the faux pas test. In Zalla et al.’s (2009) study, the examiner sat in front of the participant and read each story aloud. The story was also placed in front of the participant so they could read along themselves and remained there throughout the reading and questioning. This procedure places a large demand upon verbal auditory skill and reading comprehension. It also perhaps places a substantial demand upon imagination and imagery. We sought to try to reduce these potentially confounding variables by using a simple cartoon-like presentation of the social scenarios.

## Method

### Participants

Forty-three TD adults and thirty-five high-functioning individuals with a diagnosis on the autism spectrum took part, all of whom were native English speakers and none of whom had significant hearing, visual or motor impairments. The UCL Research Ethics Committee approved the study and written informed consent was obtained from all participants. None of the TD participants had any known psychiatric or neurological conditions or any ASD diagnoses amongst their first-degree relatives. All ASD participants had previously received a clinical diagnosis of high-functioning autism (2 participants) or Asperger’s syndrome (33 participants) from a qualified clinician according to standard diagnostic criteria. We were unable to obtain written confirmation of diagnoses for 5 participants; however they were not excluded as they provided verbal confirmation of their diagnosis, met the autism diagnostic observation schedule (ADOS; Lord et al. 2000) criteria for autism spectrum or autism, and their autism spectrum quotients (AQ; Baron-Cohen et al. 2001) were above the recommended cut-off of 32. ADOS scores were available for 32 of the 35 ASD participants, 24 meeting criteria for an ASD. The eight participants whose ADOS scores fell below the cut-off were not excluded as they provided a reliable written clinical diagnosis and their AQs were above 32. Furthermore, exclusion of these participants from the ASD groups did not change the results from the cartoon faux pas test. All ASD participants had full-scale Wechsler intelligence quotients (FSIQ) >80 (WAIS-III-UK, Wechsler 1998; WASI, Wechlser 1999). In the ASD group, separate verbal (VIQ) and performance IQ (PIQ) scores were unavailable for one participant as he was tested on the two-subtest form of the WASI.

The two groups were comparable in age (*U* = 762, *p* = .924), gender [*χ*^*2*^(1) = 1.833, *p* = .176], VIQ [*t*(75) = 1.394, *p* = .167], PIQ [*t*(58.5) = .698, *p* = .488] and FSIQ [*t*(76) = .100, *p* = .920]. However, as expected the ASD group showed significantly higher AQ scores (*U* = 1313, *p* < .001; see Table 1).

**Table 1:** Participant characteristics for matched groups: mean and (SD)

	ASD (n = 35)	TD (n = 43)
Age	35.40 (10.59)	35.33 (10.54)
Gender (M:F)	24:11	23:20
VIQ^a^	117.09 (13.83)	113.16 (10.27)
PIQ^a^	109.00 (14.70)	111.07 (10.73)
FSIQ	114.17 (14.54)	113.84 (9.71)
AQ^b,^***	35.40 (8.83)	17.13 (6.78)
ADOS^c^	12 Autism	–
	12 Autism spectrum	–
	8 None	–

*ASD* Autism spectrum disorder, *TD* typically developed, *VIQ* verbal, *PIQ* performance, *FSIQ* Full-scale intelligence quotients

*** *p* < .001

^a^Data unavailable for one ASD participant

^b^Autism spectrum quotient; data unavailable for five TD participants

^c^Data unavailable for three ASD participants

To perform an item analysis, we gathered a separate “reference TD” sample (N = 69) with a mean age of 30.5 years (SD 10.4) and mean national adult reading test (NART; Nelson and Willison 1991) IQ equivalent of 115.6 (SD 6.6; this figure is based on N = 68); fifty-four percent were male. These values are not tightly matched with the “matched TD” group nor the ASD group but this is irrelevant for this psychometric analysis since we are considering only relative differences between test items, not groups or individuals.

### Materials and Procedure

The cartoon faux pas test involved 52 short cartoon stories and was inspired by Baron-Cohen’s faux pas recognition test (Baron-Cohen et al. 1999; Stone et al. 1998), which used 10 faux pas and 10 control audio-recorded and written stories. Here, eighteen cartoons showed faux pas situations, while eighteen showed non-faux pas social situations. Seven of the faux pas cartoon stories were directly adapted from Baron-Cohen et al.’s stories; the remainder were novel. Participants were required to decide whether each of these 36 cartoons was embarrassing or not; this word was presented below each cartoon followed by a question mark (i.e.: “Embarrassing?”). This differs from Baron-Cohen et al.’s task where the test question asked whether someone had said something they should not have. The participants’ ability to understand the cartoon stories was investigated by the inclusion of sixteen additional comprehension stories depicting ordinary social situations. The question appearing below these cartoons was also a yes/no question that differed from cartoon to cartoon, and focussed on story comprehension without an obvious requirement for mentalizing. There were slightly fewer comprehension items compared to the social cognition items in order to keep the administration time of the test to a minimum.

**Fig. 1:**
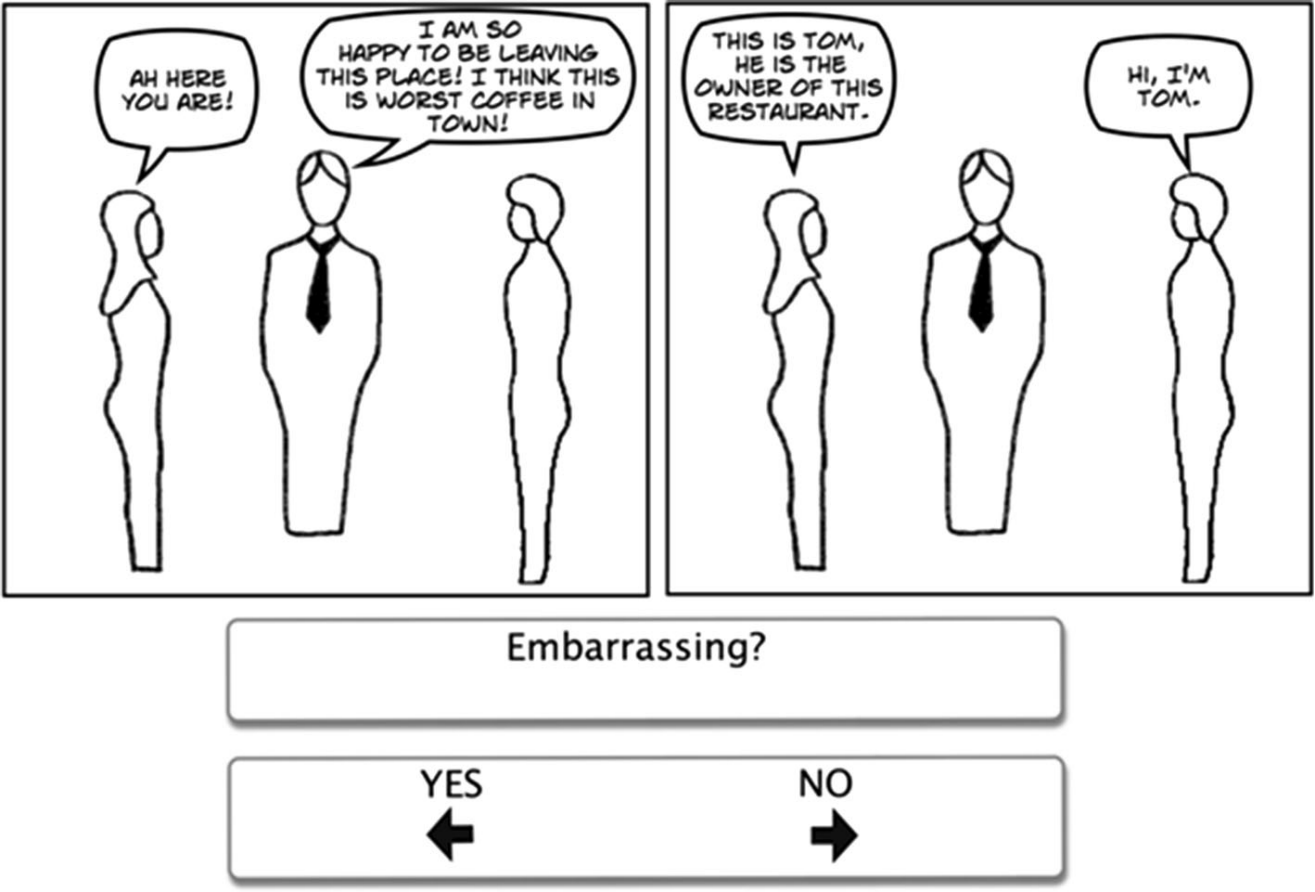
Example faux pas item

**Fig. 2:**
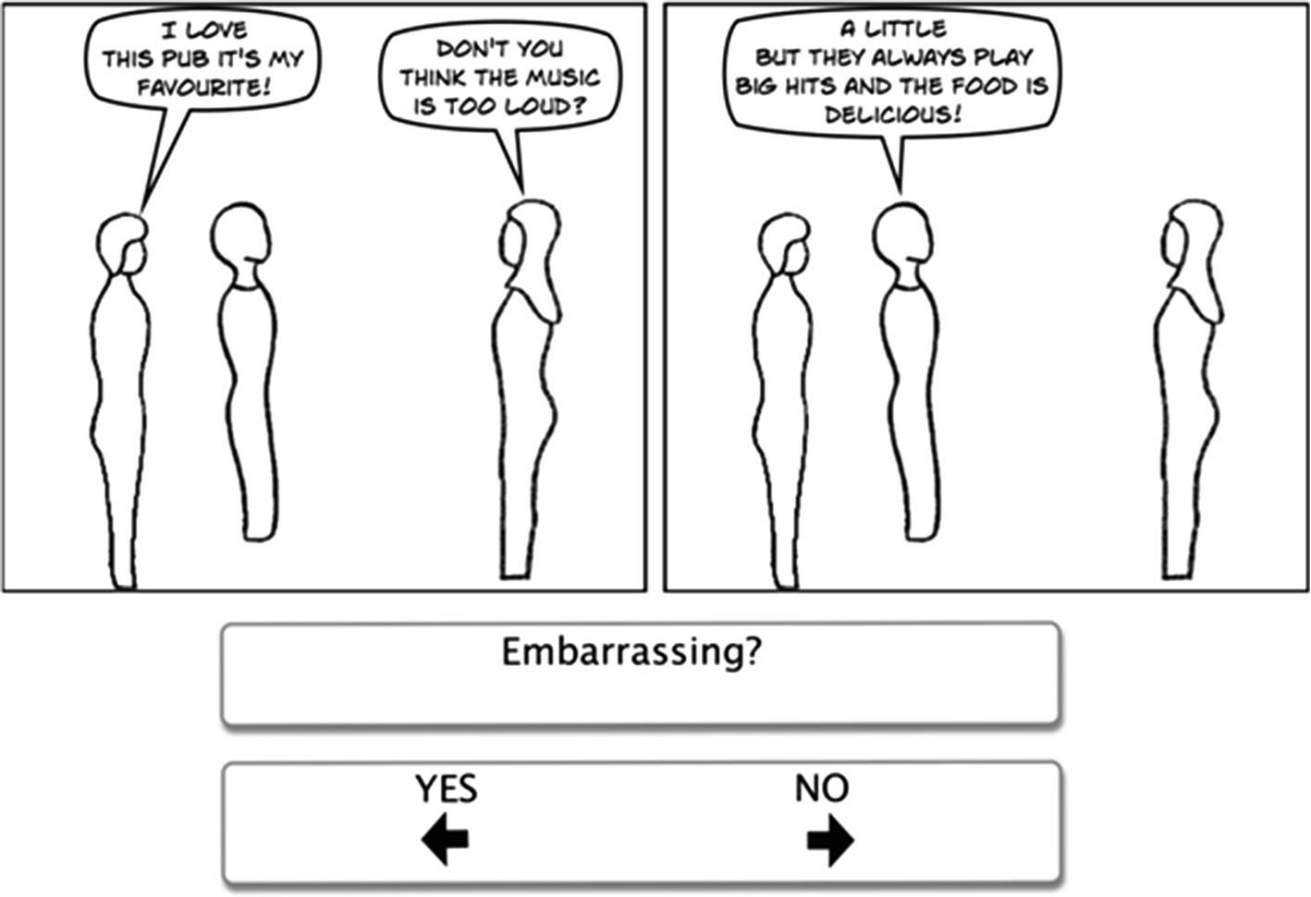
Example non-faux pas item

**Fig. 3:**
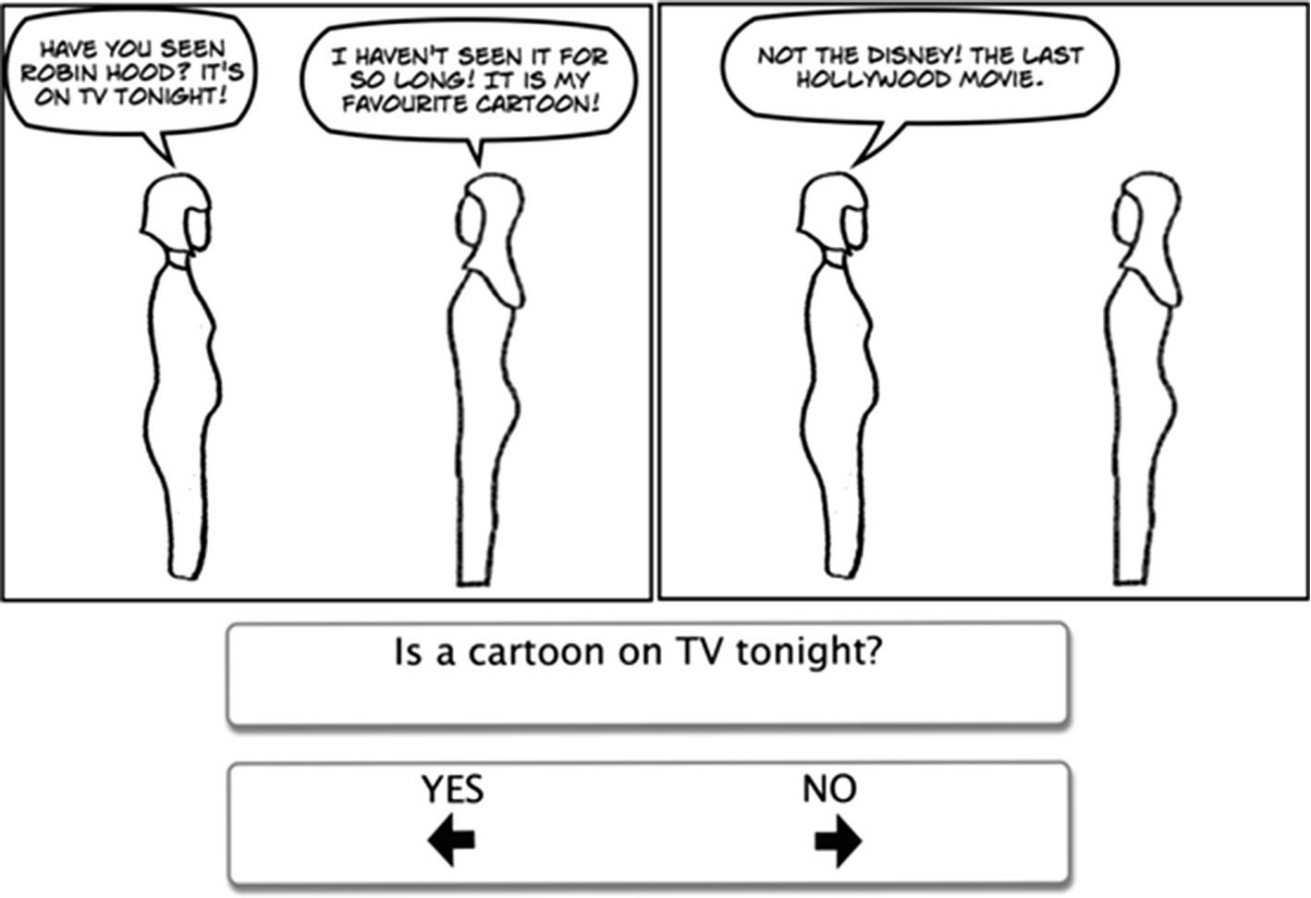
Example comprehension item

Each cartoon story involved 2 or 3 frames with an average of 30 words presented in speech bubbles (see Figs. 1, 2, 3); the exact number of words was matched strip by strip between the three cartoon types. The cartoons were simple line drawings, emphasizing the outline of the character and attempting to avoid distracting details. Each cartoon character was drawn without any facial expression, and the cartoons were created using Comic Live software (http://plasq.com/products/comiclife2/). For the faux pas cartoons, the embarrassing statement (or faux pas) occurred either in the first sentence, one of the middle sentences, or the last sentence on an equal number of occasions. The non-faux pas stories followed a similar pattern to those where a faux pas occurred, for example, someone made an error or broke into a conversation, minor incidents or quarrels occurred (e.g. children fighting, someone criticising or being disappointed), or the characters discussed serious matters (e.g. money or sickness). Every character used in the faux pas cartoons was also used in the non-faux pas cartoons.

The task was presented on a laptop using E-Prime software (Psychology Software Tools, Pittsburgh, PA, USA). Participants sat approximately 80 cm from the screen and were tested in a quiet testing room. Participants were instructed to respond as quickly as possible but without making any mistakes; a maximum of 32 s was allowed for each cartoon. Two of the comprehension cartoons were used as practice examples, followed by seven more comprehension cartoons as test items. Participants then practiced one embarrassing and one non-embarrassing cartoon, followed by the remaining 34 faux pas and non-faux pas cartoons. Lastly, seven more comprehension cartoons were presented. The order of presentation of the cartoons was randomized and held constant across participants. One of the faux pas items was excluded from the analysis because all but 2 participants answered correctly to this item so there was no useful variance in the responses. Hence, 14 comprehension, 16 faux pas and 17 non-faux pas items were included in the analyses.

### Data Analysis

The analysis was conducted in three stages. First, the accuracy performances of the matched ASD and TD groups were compared to determine if the cartoon faux pas test reliably demonstrated the previously reported pattern for a test of this type, i.e. false positive responses in the non-faux pas condition by the ASD group. Three basic scores were computed for each participant: a comprehension cartoon score (percentage of correct answers to the comprehension cartoons), a faux pas cartoon score (percentage of correct answers to cartoons containing a faux pas = true-positive answers to the “Embarrassing?” question) and a non-faux pas cartoon score (percentage of correct answers to cartoons where no faux pas was present = true-negative answers to the “Embarrassing?” question). RTs were also calculated for these three trial types. When comparing the groups’ performance, non-parametric analyses were used whenever variables violated the assumptions of normality and homogeneity.

Second, we conducted a signal detection analysis. Signal detection theory (SDT) provides a way of investigating behaviour when a person has to make a decision about whether a signal is present or not. In this case the signal is whether a faux pas has been committed. What makes SDT analysis different from standard threshold theories is that it is acknowledged that in making such a decision the participant has not only to be sensitive to a signal, but also to set a criterion for themselves as to what degree of signal will be required before they say “yes”. Specifically, SDT analysis provides two statistics. The first is called d′, and indicates the difficulty of the task for the individual. The easier the task the larger the proportion of “hits” (i.e. times when the participant has correctly said “yes” to a faux pas cartoon) and the smaller the proportion of “false positives” (i.e. times when the participant has said “yes” to a cartoon that does *not* depict a faux pas). The second statistic, called C, reflects the strategy of the participant. A participant who always says “no” will never commit a false positive. And a participant who always says “yes” will attain 100 % hits. A participant who tends to respond “yes” is called liberal and a participant who tends to respond “no” is called conservative. According to SDT, these two different measures of performance (sensitivity to the signal/detection ability; criterion-setting strategy) are theoretically independent mental components that both contribute to performance (for an overview of SDT and calculation of the appropriate statistics see Abdi 2007; Stanislaw and Todorov 1999).

In the third stage of analysis, we employed a psychometric item analysis procedure to analyse responses to individual items from the reference sample of 69 TD participants, and compared these with each of the matched groups. This allowed us to examine a standard “task difficulty” explanation for performance differences between the groups and was assessed by considering item difficulty plots. For each condition (faux-pas, non-faux pas and comprehension), the difficulty of each test item was established by considering the mean accuracy performance on that item by the N = 69 reference control sample. The mean percentage of correct responses for each of these items for the matched TD and ASD groups were then plotted against this scale. A fitted line that described the difficulty function was calculated by linear regression.

## Results

### Between-Group Comparisons

As expected, the ASD and matched TD groups correctly responded to the vast majority of comprehension control cartoons (77 %) and there was no difference between the groups (*U* = 789, *p* = .709; see Table 2). This very similar performance across the groups for the Comprehension items was not however repeated for those involving faux pas detection. Considering the faux pas and non-faux pas cartoon scores separately, TD participants correctly identified non-faux pas cartoons as not containing a faux pas 90 % of the time, whereas for ASD participants this figure was 75 %. This difference was highly significant (*U* = 342.5, *p* < .001), indicating over-detection of faux pas in the ASD group. By contrast, on average, TD participants identified faux pas cartoons as containing a faux pas 79 % of the time, while ASD participants did so for 74 % of the cartoons. This difference did not approach statistical significance (*U* = 636, *p* = .236).

**Table 2:** Accuracy and reaction times for the matched groups on the cartoon faux pas test: means (and SD)

	ASD	TD
Accuracy (% age)		
Comprehension	78 (14)	77 (12)
Faux pas	74 (17)	79 (9)
Non-faux pas	75 (19)	90 (13)
Reaction time (ms)		
Comprehension	9995 (4401)	8764 (2336)
Faux pas	11,175 (4464)	9615 (2739)
Non-faux pas	10,647 (4444)	8866 (2640)

*ASD* Autism spectrum disorder, *TD* typically developed

Given previous gender effects in ToM research (Baron-Cohen et al. 1997), it is worth noting that despite slight, non-significant disparities in gender balance between these two groups, there was no significant effect of gender on any of these measures, either across the whole sample, or within either group (all *p*s > .16).

Correlations between accuracy measures were calculated separately for each group using Spearman’s rank order correlation coefficient. In the ASD group, none of the three cartoon types related to each other (*r*_s_s < .31). In the TD group, a significant correlation was only found between the comprehension score and the faux pas item accuracy (*r*_s=_.409, *p* = .006).

Each participant’s mean RT was also measured for all three cartoon types (see Table 2). No significant difference was observed between the groups on the Comprehension cartoons [*t*(49.3) = 1.49, *p* = .142]. RTs for both groups were faster for the non-faux pas than the faux pas items [*F*(1,76) = 21.68, *p* < .001]. Across these two conditions, the ASD participants took significantly longer than the TD group to give their responses [*F*(1,76) = 4.32, *p* = .041], but there was no group by condition interaction [*F*(1,76) = .653, *p* = .421]. Unsurprisingly, RTs on the three cartoon types were significantly and positively correlated with each other in both groups (*r*s > .9, *p*s < .001).

### Signal Detection Analysis

Values of d′ and C were calculated according to Brophy (1986). The matched TD and ASD groups differed significantly in their d′ values [mean ASD 1.4 (SD .7), TD 2.0 (.5), *t* = 4.45, *p* < .001]. In other words, the ASD group were very significantly poorer at knowing if a cartoon depicted a faux pas or not. The values of C were also significantly different between the groups [TD median .15; ASD median −.11, *W* = 1919.5, *p* = .027 (adjusted for ties)]. However, a simpler way of considering the C values is just in terms of whether someone was liberal or conservative in their use of “yes” as a response across all faux pas items. According to this straightforward approach, 9 out of the 43 TD participants (20.9 %) would be classified as “liberal” in their use of “yes” as a response, whereas 20 out of 35 ASD participants (57.1 %) would be classed as liberal in use of “yes”. This difference is highly significant [*χ*^*2*^(1) = 10.833, *p* = .001].

### Item Analysis

**Fig. 4:**
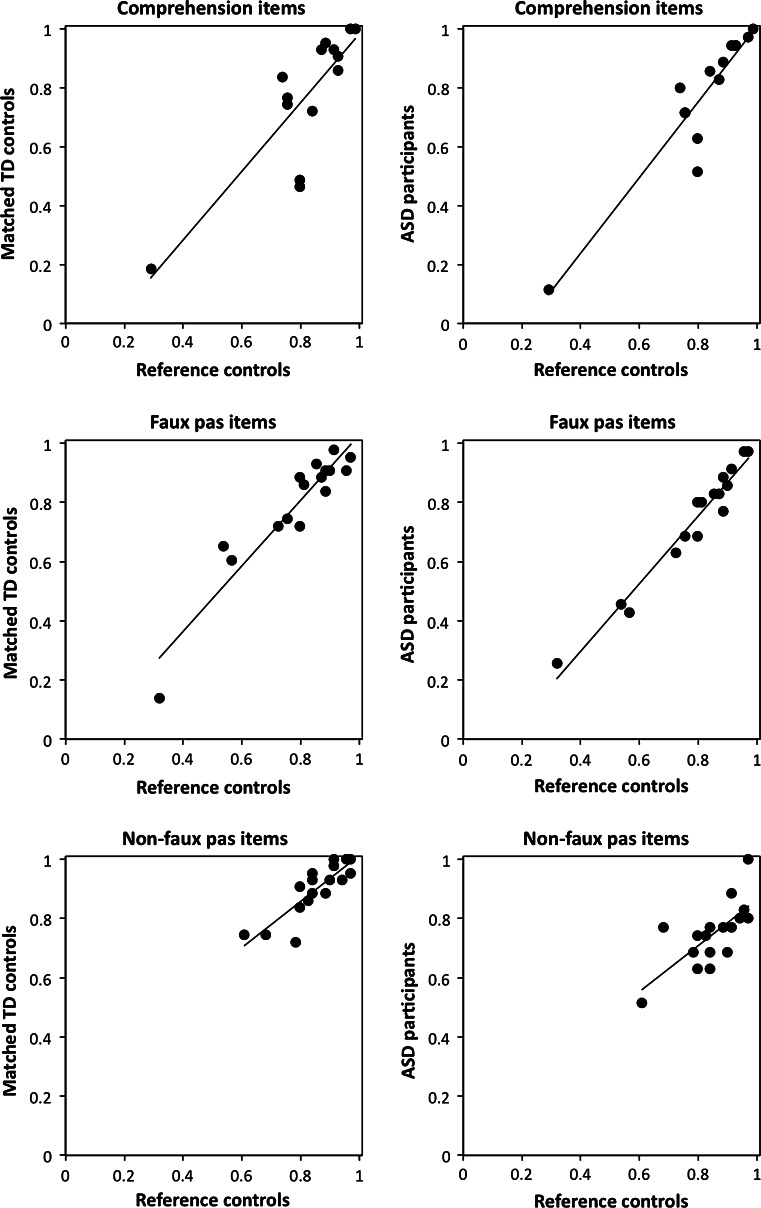
Item-by-item group accuracy for each of the three experimental conditions. The IQ- and age-matched typically-developed (TD) and autism spectrum disorder (ASD) groups are separately plotted against the performance of the reference control group. The axes show the proportion of people in each group who got a particular test item correct. *Each point* represents a particular test item. So, for instance, the figures show that when considering the performances of the faux-pas items for the ASD participants versus the reference controls, there were two test items where almost all participants in both groups got them correct, and three test items for which < 60 % of both groups got them correct

The item difficulty plots in Fig. 4 show that across all conditions, the test items that the reference group found most difficult were also those that the TD and ASD groups found most difficult (all *p* < .005). Indeed, the agreement between groups was especially high for the faux pas items, with the item-by-item correlations above .94 and the strength of the correlations was not significantly different (*z* = 1.34, *p* = .180). For the Comprehension items, the agreement was generally lower between the TD group and reference sample (*r* = .84) and between the ASD group and reference sample (*r* = .93) but the strength of these correlations was not significantly different (*z* = 1.07, *p* = .285). Likewise for the non-faux pas items, the agreement between the control group and reference sample (*r* = .86) was similar to the agreement between the ASD group and reference sample (*r* = .71; *z* = 1.07, *p* = .285). Furthermore, when comparing the strength of the correlations between the control group and reference sample, the faux pas and non-faux pas correlations did not differ (*z* = 1.21, *p* = .226). However, when comparing the strength of the correlations between the ASD group and reference sample, the faux pas correlation was significantly stronger than the non-faux pas correlation (*z* = 3.62, *p* < .001). In other words, for the ASD group only, the degree of agreement with the reference sample about item difficulty was significantly lower for the non-faux pas items than the faux pas items. For most of the test items, we could predict very well how well the ASD group would perform on each item by knowing how many of the reference sample had got them correct. But this was not the case for the non-faux pas items.

## Discussion

This study investigated the understanding of vicarious embarrassment by using a novel task, the cartoon faux pas test. High-functioning adults with clinical diagnoses on the autism spectrum were compared to typically-developed adults carefully matched on age, gender, and IQ. The key findings were the following. First, the ASD participants’ performance in answering the comprehension items (that were presented in the same cartoon format as the other items) was no different from the controls either in terms of accuracy or RT. Second, if one considers the faux pas and non-faux pas items separately the ASD participants were not significantly poorer than the IQ- and age-matched controls at detecting a faux pas when one was present (a “faux pas item”), although their mean performance was slightly below that of the controls. But when the cartoon depicted a scenario where a faux pas had *not* been committed, the ASD participants responded significantly more often that a faux pas had been. Third, for both groups of participants, RTs were correlated across the three different conditions (comprehension, faux pas and no-faux pas). Fourth, RTs for both groups were faster for the non faux-pas items than the faux pas items. Fifth, the ASD participants took significantly longer to give their responses in both faux pas and non-faux pas conditions, but there was no group by condition interaction. Sixth, signal detection analysis demonstrated that the ASD participants have both significantly greater difficulty knowing if a cartoon depicts a faux pas, and are also more likely to display a liberal confirmatory bias (i.e. say “yes” regardless of how good they are at detecting a faux pas if it is there). Finally, we found that while the ASD group were in good agreement with the reference control group about which comprehension and faux pas items were most difficult, they were in significantly less agreement for the non faux-pas items.

The tendency towards false positives in the non-faux pas condition found here replicates previous studies showing over-detection of embarrassment in ASD (Zalla et al. 2009; Hillier and Allinson 2002b), and shows that this effect is not an artefact of the particular type of material or method used to assess faux pas detection, since the material presented here was in a different format and most of the scenarios were different from previous versions. However, this pattern of results provides constraint upon theorising about the cause of this apparent “over-detection” of faux pas in ASD participants. Since the TD participants were carefully matched for age and IQ, and the ASD participants performed as well as they did on questions testing comprehension of events depicted in the cartoons, we can dismiss any explanation that the faux pas test differences here were an artefact of differences in general cognitive ability, or ability to attend to, or perceive, the test materials.

Instead, we consider here how well the data fits four potential explanations for these results. Hypothesis 1 was that ASD participants may show excessive attributions of mental states. This explanation would be supported by a pattern of results where ASD participants show good performance on faux pas items, but poor performance on non-faux pas items, with good agreement in terms of items difficulties with TD controls. This pattern was not observed here. While the ASD participants did perform similarly to the matched controls on the faux pas items and worse on the non-faux pas items, the items difficulty analysis showed poor agreement with the reference controls for the non-faux pas items.

Hypothesis 2 proposed an over-sensitivity to embarrassment, due either to compensation for poor mentalizing (Hypothesis 2a) or as a learned or compensatory strategy without the context of poor mentalizing (Hypothesis 2b). These accounts would be most consistent with a pattern of a liberal criterion for responding “yes” as assessed by the signal detection analysis, with relatively fast RTs to faux pas items, and either poor faux pas detection in the case of Hypothesis 2a or good faux pas detection in the case of Hypothesis 2b. The ASD participants did indeed show a liberal criterion for responding yes, as shown by the signal detection analysis. However, the SDT analysis also showed poor sensitivity to faux pas, which excludes Hypothesis 2b. In terms of RT, the ASD participants were slower with both faux pas and non-faux pas items than the TD controls. However, although there was no significant group by condition interaction, it was the case that the mean difference in overall speeds between faux pas and non-faux pas items was slightly smaller for the ASD participants (Table 2). So, while this result does not strongly support Hypothesis 2a, it is not inconsistent with it. In general, therefore, Hypothesis 2a is a reasonable fit to the results here.

Hypothesis 3 proposed that knowing that a faux pas has not been committed requires more subtle or detailed social judgement than detecting a faux pas when it is present. This account would be supported by good agreement between the ASD group and reference controls in terms of relative item difficulties for both faux pas and non-faux pas items, and slower RTs to non-faux pas items. This hypothesis is not supported by the results here. There was significantly less agreement between the ASD and reference controls as to the difficulty of the non-faux pas items than was found between the matched controls and the reference controls; this was not the case for the faux pas items. A typical explanation in psychometrics for such a circumstance would be that the non faux pas items are not measuring the same thing to the same degree in the two groups, and across the two sets of items. Moreover, RTs to non-faux pas items were actually faster than to the faux pas items. Thus Hypothesis 3 is not supported.

Hypothesis 4 was that deciding that something is not present is a more “open-ended” decision-making task than detecting something that is present, and that ASD participants may have a particular problem with open-ended tasks. This predicted slower RTs to the non-faux pas items and lack of agreement between the ASD participants and controls as to item difficulty for the non faux pas items. The latter finding was found here, but not the former: the ASD groups’ RTs to non-faux pas items were significantly faster than to the faux pas items. Thus this hypothesis receives only partial support.

In summary, this study has demonstrated that the phenomenon of apparent “over-detection” of embarrassment in faux pas tests by adults with diagnoses of autism spectrum conditions is found even when the materials being used differ greatly from the original versions of the test in terms of how they are presented, and even where the content of individual situations depicted is different. This phenomenon can be seen independently of issues to do with age, gender, or IQ. The hypothesis that best fits the patterns of data in this study is that the ASD participants have both a problem with detecting faux pas, and also that they adopt a liberal criterion for detection. In other words, in this study they appeared to default to saying “yes” when non-faux pas items were presented. It is noteworthy in this respect that the ASD participants did not say “yes” significantly more frequently than matched controls to the faux pas items. This suggests that it is a less likely explanation that they are showing a confirmation bias to the question presented in this format of test (“embarrassing?”) than it is that they are adopting a more liberal criterion for answering “yes”. The reason for the ASD adoption of a liberal threshold is beyond the scope of this paper. However one possibility may be that it is an adaptation to their relative inability to detect embarrassment or in response to having learned, or being told, that their differences in faux pas appreciation are likely to lead to false negatives. Thus this study may add to the debate concerning “compensatory” behaviours when considering psychometric test results in autism (see e.g. Brunsdon and Happé 2014; Johnson 2012).

Having a better understanding of the one’s own social difficulties may lead to a greater awareness of potentially embarrassing situations and, while this may lead to over-compensation, this may help individuals with autism to avoid making faux pas.
